# Polyoxazolines with a Vicinally Double-Bioactivated Terminus for Biomacromolecular Affinity Assessment

**DOI:** 10.3390/s21093153

**Published:** 2021-05-01

**Authors:** Florian Pinzner, Thorsten Keller, Jürgen Mut, Julian Bechold, Jürgen Seibel, Jürgen Groll

**Affiliations:** 1 Department of Functional Materials in Medicine and Dentistry, Institute of Functional Materials and Biofabrication and Bavarian Polymer Institute, University of Würzburg, Pleicherwall 2, 97070 Würzburg, Germany; florian.pinzner@fmz.uni-wuerzburg.de (F.P.); Thorsten.Keller@mail.uni-wuerzburg.de (T.K.); 2 Institute of Organic Chemistry, University of Würzburg, Am Hubland, 97070 Würzburg, Germany; juergen.mut@uni-wuerzburg.de (J.M.); julian.bechold@uni-wuerzburg.de (J.B.); seibel@chemie.uni-wuerzburg.de (J.S.)

**Keywords:** polyoxazolines, functionalization, lectin

## Abstract

Interactions between proteins and carbohydrates with larger biomacromolecules, e.g., lectins, are usually examined using self-assembled monolayers on target gold surfaces as a simplified model measuring setup. However, most of those measuring setups are either limited to a single substrate or do not allow for control over ligand distance and spacing. Here, we develop a synthetic strategy, consisting of a cascade of a thioesterification, native chemical ligation (NCL) and thiol-ene reaction, in order to create three-component polymer conjugates with a defined double bioactivation at the chain end. The target architecture is the vicinal attachment of two biomolecule residues to the α telechelic end point of a polymer and a thioether group at the ω chain end for fixating the conjugate to a gold sensor chip surface. As proof-of-principle studies for affinity measurements, we demonstrate the interaction between covalently bound mannose and ConA in surface acoustic wave (SAW) and surface plasmon resonance (SPR) experiments.

## 1. Introduction

Biomolecular recognition events, as are observed, for example, in receptor–ligand, antigen–antibody, DNA–protein, sugar–lectin or RNA–ribosome interactions, play an important role in a number of biological processes, such as cell signaling, the immune response, assembly of the extracellular matrix, or interaction of cells and bacteria with proteins [1,2,3]. Such recognition events, usually driven by a large number of different interactions taking effect all at once, constitute a highly complex mechanism, and it is usually challenging to investigate such interactions in their original biological context. The binding of lectins to carbohydrate ligands, for instance, has a high specificity, even though its binding affinity is only enhanced through the so-called multivalency effect, where multiple binding events exponentially enhance binding affinities [4,5,6]. It is thus crucial to rely on proper methods in quantitative investigation, and mimicking molecular and cellular recognition processes by designing synthetic setups or molecular model systems can lead to a better understanding of the nature of these interactions.

With several biophysical and biological assays [7], the first advances in measuring sugar–receptor interactions were performed by agglutination [8]. While some hemagglutination assays are based on kinetically driven aggregations, calorimetric methods in homogenous solutions allow for thermodynamic characterizations [9,10]. Apart from those measurements in solution, there are methods such as surface plasmon resonance (SPR) [11], surface acoustic wave spectroscopy (SAW), Langmuir [12], and quartz crystal microbalance (QCM) methods [13,14], where fixating the examined substrate to a measuring surface allows for a concise investigation of specific recognition events. The basis for such experiments relies on the creation of self-assembled monolayers on target gold surfaces, where hydrophilic polymers such as PEG are attached to the surface and their chain ends can be modified with respective substrates [15]. Such systems are used for the investigation of binding and affinity events, for example with concanavalin A (ConA) [16,17].

Non-functionalized linear polymer strands do not only aid as spacing units in this measuring setup, but they also act as an anchor of the substrate to the measuring surface. Polyethylene glycol (PEG) is the gold standard in this context [18]. However, most systems designed in this manner are either limited to a single substrate or do not allow for control over distance and spacing between different ligands. A spatially defined attachment of multiple substrates to single polymer strands therefore requires a set of expedient coupling reactions and a suitable pre-functionalization of the polymer. Promising alternative polymer candidates are polyglycerols [19] or the more flexible synthetic polyoxazolines (POx) [18,20,21].

Compared to two-component conjugates, the complexity of design strategies and synthetic challenges are increased massively when three or more different compounds are conjugated. Limitations lie within aspects of chemoselectivity of the conjugation reactions, reactivity decline, control over ligand spacing and distance, as well as steric hindrances [22]. Multicomponent reactions (MCRs) have most recently emerged as one method to ligate multiple low molecular reaction partners, usually in one-pot syntheses [23]. However, due to reactive impediments, only a few polymers have since been used for MCR, with the main focus lying on (poly)saccharides [24,25,26,27], which so far leaves this set of reactions as an unsuitable synthesis method for the aforementioned system [28].

Here, we describe a reaction sequence of three consecutive steps that lead to the formation of a novel three-component polymer conjugate with a defined architecture, which in turn can be used in self-assembled monolayer affinity measuring setups. It enables an interaction of two bound substrates with larger biocomponents, while maintaining a fixed distance between the different ligands. First, the relevant polymer educts, α thioester and ω thioether functionalized polyoxazolines, are synthesized. While the thioester group is used for subsequent coupling reactions, the thioether group with its high affinity to gold [29] acts as an “anchor” for the fixation of the conjugate to the sensor gold surface. Secondly, the native chemical ligation (NCL), which is one of the major and commonly used methods in peptide ligation, are used to couple the thioester functionalized polymers with cysteine functionalized peptides. A free thiol group emerging from the NCL can in turn be used to chemoselectively attach an allyl component via a thiol-ene reaction, which is perceived as a very reliable click reaction [30,31].

The success of all cascade reaction steps leading to the final conjugation product was proven through biomacromolecular recognition experiments. As bioactive compounds, carbohydrates were implemented and the conjugate’s interaction with lectins was evaluated by performing affinity measurements via two different methods: SAW and SPR spectroscopy.

## 2. Materials and Methods

### 2.1. Materials

Reagents and solvents were, unless stated otherwise, commercially available as reagent grade, and did not require further purification. As solvents, acetic acid (AcOH) (30%, Sigma Aldrich), acetone (≥99.5%, Sigma Aldrich, Darmstadt, Germany), acetonitrile (≥99.9%, Honeywell, Charlotte, USA), chloroform (CHCl_3_) (≥99.8%, Fisher, Schwerte, Germany), dichloromethane (DCM) (≥99.9%, Sigma Aldrich), diethyl ether (Et_2_O) (≥98%, Sigma Aldrich), dimethyl sulfoxide (DMSO) (≥98%, Sigma Aldrich), methanol (MeOH) (≥98%, Sigma Aldrich), and tetrahydrofuran (THF) (≥98%, Sigma Aldrich) were used as received. As reagents, 2,2-dimethoxy-2-phenylacetophenone (DMPA) (99%, Sigma Aldrich), 2-hydroxy-4′-(2-hydroxyethoxy)-2-methylpropiophenone (Irgacure 2959) (98%, Sigma Aldrich, BASF, Ludwigshafen am Rhein, Germany), 3-mercaptopropionic acid (≥99%, Sigma Aldrich), 4 Å molecular sieve (Sigma Aldrich), allyl alcohol (98%, Sigma Aldrich), allylamine (98%, Sigma Aldrich), benzyltrimethylammonium chloride (98%, Sigma Aldrich), calcium hydride (92%, abcr GmbH, Karlsruhe, Germany), dialysis membranes (Spectrum Laboratories, New Brunswick, NJ, USA), ethanethiol (98%, Sigma Aldrich), isobutyl chloroformate (98%, Sigma Aldrich), magnesium sulfate (≥99.5%, Sigma Aldrich), *N*-methylmorpholine (≥98.0%, Fisher, Schwerte, Germany), sodium borohydride (99%, Acros Organics), sodium hydroxide (≥99%, Sigma Aldrich, Darmstadt, Germany), potassium hydroxide (≥99%, Merck), thiophenol (≥99%, Sigma Aldrich, AlphaAesar, Kandel, Germany), tosyl chloride (98%, Sigma Aldrich), tris(2-carboxyethyl)phosphine (TCEP) (≥98%, Sigma Aldrich, Roth) and tris(hydroxypropyl)phosphine (THPP) (80%, abcr GmbH) were used as received.

All peptides (CGGGF, CKFKFQF and CGGWYKYW) were ordered from *GeneCust* and used as received. Recombinant Concanavalin A (ConA) (≥90%, *Aldrich*) were used as received.

Acetonitrile (99.9%, Sigma-Aldrich), 2-methyl-2-oxazoline (MeOx) (98%, Sigma-Aldrich) and *p*-toluenesulfonate (98%, Sigma Aldrich) for polymer synthesis were dried over CaH_2_ and distilled before use.

### 2.2. Methods

**FT-IR** spectroscopy was performed with an instrument by Thermo Scientific, carrying an ATR unit and a deuterated triglycine sulfate—potassium bromide (DTGS-KBr) detector, covering a wavenumber range from 4000 cm^−1^ to 650 cm^−1^.

Microwave reactions were performed with a microwave (20–300 °C, max. 300 watt) by *CEM GmbH*. Depending on the reaction mixture volume, either 10 mL or 35 mL glass vials with a silicone cap were used.

**NMR** spectroscopy was performed with an instrument by *Bruker* at 300 MHz with 128 scans. Chemical shifts *δ* are given in ppm. The ^1^H resonance signal of the used solvent was used as internal reference for ^1^H-NMR spectra (deuterated acetonitrile (CD_3_CN-*d3*): 1.94 ppm; deuterated chloroform (CDCl_3_): 7.26 ppm; deuterated dimethyl sulfoxide (DMSO-*d6*): 2.50 ppm; deuterated methanol (MeOD): 3.31 ppm; deuterated water (D_2_O): 4.79 ppm).

**SEC** measurements in DMF were performed on an instrument from Malvern Instruments with an oven temperature of 45 °C, DMF with 1 g·L^−1^ lithium bromide as eluent and an applied flow rate of 1.0 mL·min^−1^. The system includes a high-pressure liquid chromatography pump (Agilent), an autosampler, and a precolumn (Dguard, Organic Guard Column, length: 10 mm, width: 4.6 mm), as well as an SEC column D2000 for organic solvents, (length: 300 mm, width: 7.8 mm, styrene-divinylbenzene (styrene-DVB) polymer, particle size: 6 µm, exclusion limit: 5000 g·mol^−1^), and SEC column D3000 for organic solvents (length: 300 mm, width: 7.8 mm, styrene-divinylbenzene (styrene-DVB) polymer, particle size: 6 µm, exclusion limit: 70,000 g·mol^−1^). As detectors, a refractive index detector (Agilent), a viscosity detector (Agilent), a right-angle light scattering detector (Agilent), and a low-angle light scattering detector (Agilent) were used. For conventional calibration, 1–65 kDa PEG standards by Malvern were used. For sample preparation, 5 mg of the samples were dissolved in 1 mL of the eluent and filtered with PTFE filters with a 0.2 µm pore size.

**SAW** measurements were performed with apparatus from SAW Instruments GmbH, which included an autosampler and allowed for measuring of protein adsorption and K_D_ value determination. Measurements were performed at 25 °C using PBS as a running buffer. For the immobilization of polymers containing a thioether group on the bare gold sensor chip surface 30 µL of polymers per lane (concentration: 10 µg·mL^−1^ in PBS) were preincubated onto the sensor chip for 1 h with an external application mask. Remaining unspecific protein binding sites on the bare gold surface were saturated by the injection of 500 µM BSA. Purified recombinant ConA was used as an analyte at four concentrations, ranging from 118 to 944 nM. The flow rate for the acquisition of interaction data was set to 40 µL·min^−1^ in all experiments. Association was measured for 300 s, then dissociation was initiated by perfusing the running buffer, and the dissociation phase was also monitored for 300 s. Regeneration of the chip surface was performed by injection of two 60 s pulses of 100 mM glycine (pH 2.5) at a flow rate of 100 µL·min^−1^. Interaction data were analyzed using the FitMaster add-in (SAW Instruments GmbH) of Origin software version 8.5 (OriginLab Corporation), applying a simple Langmuir-type 1:1 interaction model. Association rate constant k_on_ values and dissociation rates constant k_off_ values were obtained by fitting data of individual experiments. Equilibrium binding K_D_ values were deduced using the equation K_D_ = k_off_/k_on_. The sensor chips integrate 5 independent sensor elements (lanes) and allow for the simultaneous analysis of different species or parameters and parallel references. All SAW experiments were performed in at least three independent experiments.

Interactions between polymers and Concanavalin A (ConA) were measured by **SPR** using a REICHERT^®^4SPR system (Reichert Technologies). Measurements were performed at 25 °C using HBS150T (10 mM HEPES (pH 7.4), 150 mM NaCl, 3.4 mM EDTA, 0.005% (*v*/*v*) Tween 20) as running buffer. For the immobilization of polymers containing a thioether group on the bare gold sensor chip surface, the polymers were injected onto a sensor for 8 min at a flow rate of 10 µL/min to cause polymer coating to a final density of 1000 resonance units. Remaining unspecific protein binding sites on the bare gold surface were saturated by the injection of 500 µM BSA. Purified recombinant ConA was used as an analyte at four concentrations, ranging from 118 to 944 nM. The flowrate for the acquisition of interaction data was set to 25 µL·min^−1^ in all experiments. Association was measured for 180 s, then dissociation was initiated by perfusing running buffer, and the dissociation phase was monitored for 300 s. Regeneration of the chip surface was performed by injecting two 60 s pulses of 100 mM glycine (pH 2.5) at a flow rate of 100 µL·min^−1^. Interaction data were analyzed using the software TraceDrawer version 1.8.1 (Ridgeview Instruments AB, Uppsala, Sweden), applying a simple Langmuir-type 1:1 interaction model and using global fitting for the rate constants. Association rate constant k_on_ values and dissociation rates constant k_off_ values were obtained by fitting the data of individual experiments. Equilibrium binding K_D_ values were deduced using the equation K_D_ = k_off_/k_on_. All SPR experiments were performed in at least three independent experiments.

**Titration** experiments were performed with a titrator from Metrohm with two Metrohm 800 Dosino dosing units and a pH electrode for small sample volumes (Metrohm Biotrode). For each measurement, 25 mg samples were dissolved in 2 mL water and titrated against 0.01 m respective 0.001 m NaOH solution.

**Photoinitiated reactions** were performed with three UV LED cubes by Polymerschmiede GmbH with an output of 11 W (each) and a wavelength of 365 nm.

### 2.3. Synthesis

Synthesis of allyl *p*-Toluenesulfonate.

The synthesis of allyl *p*-Toluenesulfonate was oriented to synthesis procedures that have been described elsewhere [32,33,34]. Results of **^1^H-NMR** (CDCl_3_, 300 MHz): *δ* 7.78 (d, *J* = 8.3 Hz, 2H), 7.33 (d, *J* = 8.0 Hz, 2H), 5.87–5.74 (m, 1H), 5.34–5.21 (m, 2H), 4.52 (d, *J* = 5.9 Hz, 2H), 2.43 (s, 3H) ppm.

Synthesis of α and ω telechelic allyl and thioether functionalized allyl-PMeOx_20_-SEt.

Under argon atmosphere, 424 µL (500 mg, 2.36 mmol, 1.00 eq.) dried allyl *p*-toluenesulfonate were dissolved in 10 mL acetonitrile and 3.99 mL (4.00 g, 80.5 mmol, 50.0 eq.) 2-methy-2-oxazoline were added. The reaction mixture was stirred for 1 h at 100 °C in a microwave reactor. Afterwards, 524 µL (440 mg, 7.08 mmol, 3.00 eq.) ethanethiol were added and the reaction mixture was stirred at 40 °C for 48 h. The raw product was precipitated four times in 100 mL cold diethyl ether. Between the precipitation steps, solvent was decanted, and the residue was taken up in 5 mL MeOH/CHCl_3_. After the final precipitation, solvent was removed in vacuo. Results of **^1^H-NMR** (CDCl_3_, 300 MHz): *δ* 7.62 (d, tosylate counterion), 7.13 (d, tosylate counterion), 5.79–5.68 (m, 1H), 5.22–5.07 (m, 2H), 3.92 (m, 2H), 4.28 (s, 2H), 3.87–3.41 (br. m, 80H), 2.82 (s, 4H), 2.31 (s, tosylate counterion), 2.09–1.99 (br. m, 63H); **FT-IR** (ATR): *ῦ* = 3444 (br. w), 2937 (w, ν(C–H)), 1743 (w), 1626 (vs, ν(C=O)), 1477 (w), 1416 (vs), 1363 (m), 1235 (m), 1120 (w), 1032 (w), 1010 (m), 926 (w), 819 (w), 749 (m), 680 (w) cm^−1^.

Synthesis of α telechelic carboxylic acid functionalized COOH-PMeOx_20_-SEt.

For this synthesis, 2.50 g (1.39 mmol, 1.00 eq.) allyl functionalized allyl-PMeOx_20_-SEt and 362 µL (441 mg, 4.16 mmol, 3.00 eq.) 3-mercaptopropionic acid were dissolved in 5 mL MeOH. A total of 178 mg (0.67 mmol, 0.50 eq.) DMPA were added, and the reaction mixture was stirred for 60 min under UV light irradiation. The raw product was precipitated three times in 100 mL cold diethyl ether. Between the precipitation steps, solvent was decanted, and the residue was taken up in 5 mL MeOH/CHCl_3_. After the final precipitation, solvent was removed in vacuo. Results of **^1^H-NMR** (CDCl_3_, 300 MHz): *δ* 7.67 (tosylate counter ion), 7.18 (tosylate counter ion), 4.33 (s, 2H), 3.46 (br. m, 80H), 2.79–2.71 (m, 4H), 2.71–2.59 (m, 4H), 2.36 (s, tosylate counterion), 2.14–2.07 (br. m, 63H), 1.86 (s, 2H); **FT-IR** (ATR): *ῦ* = 3439 (br. w, ν(N–H)), 2939 (w, ν(C–H)), 1735 (m, ν(C=O, COOH)), 1619 (vs, ν(C=O)), 1479 (w), 1417 (s), 1364 (m), 1212 (m), 1121 (w), 1032 (m), 1010 (m), 819 (w), 753 (m), 681 (w) cm^−1^.

Synthesis of α telechelic thioester functionalized COSPh-PMeOx_20_-SEt.

In a flame-dried flask, 2.00 g (1.05 mmol, 1.00 eq.) carboxylic acid functionalized COOH-PMeOx_20_-SEt and 138 µL (127 mg, 1.26 mmol, 1.20 eq.) *N*-methylmorpholine were dried for 90 min over a 4 Å molecular sieve in 15 mL chloroform and 5 mL THF. Then, 150 µL (157 mg, 1.15 mmol, 1.10 eq.) isobutylchloroformate were added and the reaction mixture was stirred for another 15 min. Next, 129 µL (138 mg, 1.26 mmol, 1.20 eq.) thiophenol were added, followed by another 138 µL (127 mg, 1.26 mmol, 1.20 eq.) *N*-methylmorpholine and the reaction mixture was stirred at RT overnight. The reaction mixture was filtered to remove molecular sieves and the raw product was precipitated three times in 100 mL cold diethyl ether. Between the precipitation steps, solvent was decanted, and the residue was taken up in 5 mL CHCl_3_. After the final precipitation, solvent was removed in vacuo. Results of **^1^H-NMR** (CDCl_3_, 300 MHz): *δ* 7.71 (tosylate counter ion), 7.40 (s, 5H), 7.18 (tosylate counter ion), 4.37 (s, 2H), 4.13 (t, isobutyl chloroformate by-product), 3.96 (d, isobutyl chloroformate by-product), 3.46 (br. s, 80H), 2.93–2.91 (m, 4H), 2.84 (s, isobutyl chloroformate by-product), 2.55–2.53 (m, 4H), 2.35 (tosylate counter ion), 2.13–2.06 (br. m, 63H), 1.85 (s, 2H); **FT-IR** (ATR): *ῦ* = (br. w, ν(N–H)), 2939 (w, ν(C–H)), 1737 (w, (C=O), COSPh), (1621 (vs, ν(C=O)), 1478 (w), 1417 (vs), 1364 (m), 1237 (m), 1115 (m), 1032 (m), 1010 (m), 753 (w), 682 (w) cm^−1^.

Synthesis of α telechelic peptide functionalized polymer conjugates CGGGF-PMeOx_20_-SEt and CGGWYKYW-PMeOx_20_-SEt.

The synthesis of polymer peptide conjugates CGGGF-PMeOx_20_-SEt and CGGWYKYW-PMeOx_20_-SEt followed a standardized procedure: 1.00 eq. of the peptide were dissolved in MeOH. Additionally, 1.00 eq. thioester functionalized polymer were dissolved in MeOH and added to the peptide solution. Then, 2.00 eq. sodium borohydride were added. Gas formation and heat development was observed. The reaction mixture was stirred for 24 h at RT for the CGGGF peptide, and at 65 °C for the less-soluble CGGWYKYW peptide. The raw product was dialyzed against water (cut-off: 1000 g·mol^−1^) for 4 d. After dialysis, solvent was removed via lyophilization. **CGGGF-PMeOx_20_-SEt: ^1^H-NMR** (DMSO-*d6*, 300 MHz): *δ* 8.10–7.29 (br. m, NH/NH_2_), 7.19 (s, 5H), 4.55 (s, 1H), 4.32 (s, 2H), 4.09–3.70 (m, 6H), 3.35 (br. s, 4nH), 3.03–2.57 (m, 13H), 2.00–1.97 (br. m, 3nH+3), 1.80–1.77 (m, 2H); **FT-IR** (ATR): *ῦ* = 3448 (br. w, ν(N–H)), 3305 (w, ν(O–H)), 2937 (w, ν(C–H)), 1736 (w), 1626 (vs, ν(C=O)), 1550 (w, (ν(N–H) peptide amide), 1478 (w), 1417 (s), 1364 (w), 1324 (w), 1240 (m), 1213 (w), 1013 (m), 980 (w), 928 (w), 758 (w), 702 (w) cm^−1^. **CGGWYKYW-PMeOx_20_-SEt: ^1^H-NMR** (DMSO-*d6*, 300 MHz): *δ* 10.71 (d, tryptophan NH), 9.11–7.90 (br. m, NH/NH_2_), 7.56–6.56 (m, tryptophan and tyrosine aromatic rings), 4.48–3.60 (m, peptide backbone), 3.34 (br. s, DMSO, polymer backbone), 3.07–2.66 (m, polymer alkyl chain CH_2_, peptide side chain CH_2_), 1.99–1.97 (br. m, polymer side chain methyl groups, polymer CH_3_ ω end group), 1.41–0.87 (m, lysine side chain CH_2_); **FT-IR** (ATR): *ῦ* = 3282 (br. m, ν(O–H)), 2938 (w, ν(C–H)), 1732 (m), 1615 (vs, ν(C=O)), 1515 (m, ν(N–H) peptide amide), 1482 (w), 1419 (s), 1363 (m), 1238 (m), 1122 (w), 1033 (m), 1011 (m), 931 (w), 801 (w), 745 (m), 682 (m) cm^−1^.

Synthesis of 1,2,3,4,6-Penta-*O*-acetyl-α-d-mannopyranoside.

D-Mannose (2.00 g, 11.2 mmol) was suspended in acetic anhydride (40.0 mL, 423 mmol, 38.0 eq.) and treated with catalytic amounts of conc. Sulfuric acid (200 µL). The mixture was stirred for 2.5 h at RT before it was diluted with CH_2_Cl_2_ (80 mL) and washed with H_2_O (20 mL), sat. NaHCO_3_ solution (20 mL), and brine (20 mL). Organic phases were dried over Na_2_SO_4_ and solvent was evaporated under reduced pressure to yield a colorless oil as an anomeric mixture with α/β 14:1 (4.37 g, 11.2 mmol, quant.). **R_f_-value:** 0.80 (CH_2_Cl_2_/MeOH 3:1); **^1^H-NMR** (CDCl_3_, 400 MHz): *δ* 6.08 (d, 1H, ^3^*J* = 1.6 Hz), 5.35–5.33 (m, 2H, H-4), 5.26–5.25 (m, 1H), 4.27 (dd, 1H, ^3^*J* = 4.9 Hz, ^2^*J* = 12.4 Hz), 4.08 (dd, 1H, ^3^*J* = 2.4 Hz, ^2^*J* = 12.4 Hz), 4.06–4.02 (m, 1H), 2.17, 2.17, 2.09, 2.04, 2.00 (s, 5 × 3H, 5 × C(O)C*H*_3_) ppm; **^13^C-NMR** (CDCl_3,_ 100.6 MHz): *δ* 170.8, 170.1, 169.9, 169.7, 168.2 (5C, 5 × *C*(O)CH_3_), 90.7 (*C*-1), 70.7 (*C*-5), 68.8 (*C*-3), 68.4 (*C*-2), 65.6 (*C*-4), 62.2 (*C*-6), 21.0, 20.9, 20.8, 20.8, 20.8 (5C, 5 × C(O)*C*H_3_) ppm.

Synthesis of 2,3,4,6-Tetra-*O*-acetyl-1-*O*-allyl-α-d-mannopyranoside.

For this reaction, 2,3,4,6-Tetra-*O*-acetyl-1-*O*-allyl-α-d-mannopyranoside (2.00 g, 5.12 mmol) was dissolved in anhydrous CH_2_Cl_2_ (40 mL) and treated with allyl alcohol (501 µL, 8.20 mmol, 1.60 eq.). Subsequently, BF_3_*Et_2_O (949 µL, 7.79 mmol, 1.50 eq.) was added dropwise, and the mixture was refluxed for 48 h. Afterwards, NEt_3_ (8 mL) was added before the mixture was diluted with CH_2_Cl_2_ (40 mL) and washed with sat. NaHCO_3_ solution (40 mL). The aqueous phase was extracted with CH_2_Cl_2_ (3 × 30 mL), the combined organic phases were dried over Na_2_SO_4_, and solvent was evaporated under reduced pressure. Purification by column chromatography (Cy/EE 1:1) yielded the product as a colorless oil (1.41 g, 3.63 mmol, 70%). **R_f_-value:** 0.40 (Cy/EE 1:1); **^1^H-NMR** (CDCl_3_, 400 MHz): *δ* 5.94–5.84 (m, 1H,), 5.37 (dd, 1H, ^2^*J* = 10.0, 3.48 Hz), 5.33–5.21 (m, 4H), 4.86 (d, 1H, ^3^*J* = 1.8 Hz), 4.28 (dd, 1H, ^3^*J* = 5.3 Hz, ^2^*J* = 12.2 Hz), 4.19 (dddd, 1H, ^2^*J* = 12.8 Hz, ^3^*J* = 5.2 Hz, ^4^*J* = 1.4 Hz), 4.10 (dd, 1H, ^3^*J* = 2.4 Hz, ^2^*J* = 12.2 Hz), 4.05–3.99 (m, 2H), 2.15, 2.10, 2.04, 1.99 (s, 4 × 3H, 4 × C(O)C*H*_3_) ppm; **^13^C-NMR** (CDCl_3_, 100.6 MHz): *δ* 170.8, 170.2, 170.0, 169.9 (4C, 4 × *C*(O)CH_3_), 133.0 (*C*-2′), 118.6 (*C*-3′), 96.7 (*C*-1), 69.8 (*C*-2), 69.2 (*C*-3), 68.8 (*C*-1′), 68.7 (*C*-5), 66.6 (*C*-4), 62.6 (*C*-6), 21.1, 20.9, 20.9, 20.8, (4C, 4 × C(O)*C*H_3_) ppm.

Synthesis of 1-*O*-allyl-α-d-mannopyranoside.

For this synthesis, 2,3,4,6-Tetra-*O*-acetyl-1-*O*-allyl-α-d-mannopyranoside (1.00 g, 2.57 mmol) was suspended in anhydrous methanol (10 mL). The addition of NaOMe (5.15 mL, 0.5 m in MeOH, 2.57 mmol, 1.00 eq.) yielded a clear solution that was stirred for 15 min at RT. The mixture was neutralized with Amberlite IR 125, the cation exchanger was filtered off, and the solvent was evaporated. The product was isolated as a colorless solid (545 µg, 2.47 mmol, 96%). **R_f_-value:** 0.10 (DCM/MeOH 15:1); **^1^H-NMR** (MeOD, 400 MHz): *δ* 5.98–5.89 (m, 1H), 5.29 (dddd, 1H, ^3^*J* = 17.2, ^2^*J* = 3.4 Hz, ^4^*J* = 1.8 Hz), 5.17 (dddd, 1H, ^3^*J* = 10.5, ^2^*J* = 3.4 Hz, ^4^*J* = 1.4 Hz); 4.79 (d, 1H, ^3^*J* = 1.7 Hz), 4.21 (dddd, 1H, ^2^*J* = 13.1 Hz, ^3^*J* = 5.1 Hz, ^4^*J* = 1.4 Hz), 4.00 (dddd, 1H, ^2^*J* = 13.1 Hz, ^3^*J* = 5.8 Hz, ^4^*J* = 1.4 Hz), 3.85–3.80 (m, 2H), 3.72–3.69 (m, 2H), 3.63–3.58 (m, 1H), 3.55–3.52 (m, 1H) ppm; **^13^C-NMR** (MeOD, 100.6 MHz): *δ* 135.5 (*C*-2′), 117.2 (*C*-3′), 100.7 (*C*-1), 74.7 (*C*-5), 72.6 (*C*-3), 72.2 (*C*-2), 68.8 (*C*-1′), 68.6 (*C*-4), 62.9 (*C*-6) ppm.

Synthesis of α telechelic sugar and peptide functionalized polymer conjugates Man-CGGGF-PMeOx_20_-SEt, AcMan-CGGWYKYW-PMeOx_20_-SEt and Man-CGGWYKYW-PMeOx_20_-SEt.

The synthesis of polymer peptide sugar conjugates Man-CGGGF-PMeOx_20_-SEt, AcMan-CGGWYKYW-PMeOx_20_-SEt, and Man-CGGWYKYW-PMeOx_20_-SEt followed a standardized procedure: 1.00 eq. peptide polymer conjugate and 0.5–2 eq. TCEP·HCl were dissolved in a 1:1–2 mixture of D_2_O and MeOD, resp. DMSO, depending on the solubility of the conjugate. Then, 1.00 eq. sugar and 0.60 eq. Irgacure 2959 were added. The reaction mixture was stirred for 60 min under UV light irradiation. The reaction mixture was dialyzed against water for 4 d (cut-off: 1000 g·mol^−1^) and dried via lyophilization. **Man-CGGGF-PMeOx_20_-SEt:** **^1^H-NMR** (MeOD, 300 MHz): *δ* 8.15–7.08 (br. m), 4.66 (m), 4.57 (s), 4.23–3.72 (m), 3.53 (br. s), 3.05–2.59 (m), 2.15–2.05 (br. m), 1.94–1.87 (m); **FT-IR** (ATR): *ῦ* = 3425 (br. w, v(N–H)), 2938 (w, ν(C–H)), 1734 (w), 1624 (vs, ν(C=O)), 1544 (w, ν(N–H) peptide amide), 1479 (w), 1418 (vs), 1364 (m), 1318 (w), 1241 (m), 1211 (w), 1083 (w), 1013 (m), 926 (w), 760 (w), 700 (m) cm^−1^. **AcMan-CGGWYKYW-PMeOx_20_-SEt: ^1^H-NMR** (DMSO-*d6*, 300 MHz): *δ* 10.74 (s, tryptophan NH), 9.12–7.99 (br. m, NH/NH_2_), 7.56–6.59 (m, tryptophan and tyrosine aromatic rings), 5.14–5.10 (m, sugar ring), 4.48–3.60 (m, peptide backbone), 3.33 (br. s, DMSO, polymer backbone), 2.83–2.60 (m, polymer alkyl chain CH_2_, peptide side chain CH_2_, sugar CH_2_ chain), 1.99–1.78 (br. m, polymer side chain methyl groups, polymer CH_3_ ω end group, sugar acetyl signals), 1.23–0.87 (m, lysine side chain CH_2_); **FT-IR** (ATR):*ῦ* = 3402 (br. w, v(N–H)), 3291 (br. w, v(O–H)), 2938 (w, ν(C–H)), 1736 (w, ν(C=O) acetyl sugar)), 1620 (vs, ν(C=O)), 1516 (w, ν(N–H) peptide amide), 1480 (w), 1419 (vs), 1365 (m), 1322 (w), 1241 (s), 1034 (w), 1014 (m), 927 (w), 751 (m) cm^−1^. **Man-CGGWYKYW-PMeOx_20_-SEt: ^1^H-NMR** (DMSO-*d6*, 300 MHz): *δ* 10.72 (s, tryptophan NH), 9.10–7.98 (br. m, NH/NH_2_), 7.98–6.57 (m, tryptophan and tyrosine aromatic rings), 4.59 (anomeric sugar signal), 4.48–3.60 (m, peptide backbone), 3.91–3.77 (m, sugar ring), 3.34 (br. s, DMSO, polymer backbone), 2.86–2.61 (m, polymer alkyl chain CH_2_, peptide side chain CH_2_, sugar CH_2_ chain), 2.01–1.97 (br. m, polymer side chain methyl groups, polymer CH_3_ ω end group, sugar acetyl signals), 1.80–0.87 (m, lysine side chain CH_2_); **FT-IR** (ATR): *ῦ* = 3281 (br. w, ν(O–H)), 2938 (w, ν(C–H)), 1729 (w), 1622 (vs, ν(C=O)), 1516 (w, ν(N–H) peptide amide), 1480 (w), 1419 (s), 1364 (m), 1315 (w), 1240 (m), 1101 (w), 1032 (w), 1013 (m), 922 (w), 804 (w), 748 (m), 700 (w) cm^−1^.

## 3. Results

### 3.1. Overall Aim and Strategy

**Figure 1:**
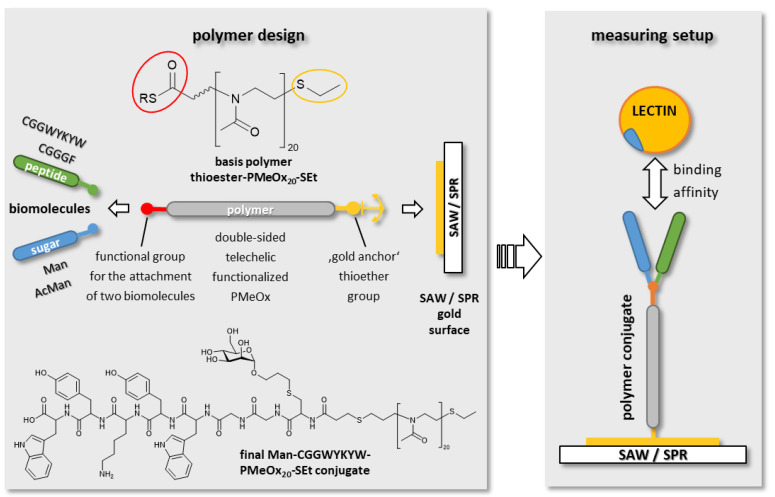
Simplified depiction of polymer design and measuring setup. SAW: surface acoustic wave spectroscopy. SPR: surface plamon resonance spectroscopy.

We provide a stepwise synthetic strategy to create novel three-component polymer conjugates with a defined architecture that can be used in self-assembled monolayer affinity measuring setups. The target design is the attachment of two functional “arms” to the α telechelic end of a polymer (see Figure 1), whereby control over the relative space between both ligands is obtained by attaching them vicinal at one binding site.

The success of all cascade reaction steps leading to the final conjugation product was proven through biomacromolecular recognition experiments. Although one specific targeted interaction was the focus of this work, the affinity between mannose and ConA, the measuring setup was developed and designed with the intention of its capability being extended to display multivalent binding modes. Although it exhibited no specific biological interaction in this setting, a proof-of-principle feasibility to introduce a second biological moiety was demonstrated by attaching different peptide molecules in addition to the glycoside unit.

**Scheme 1:**
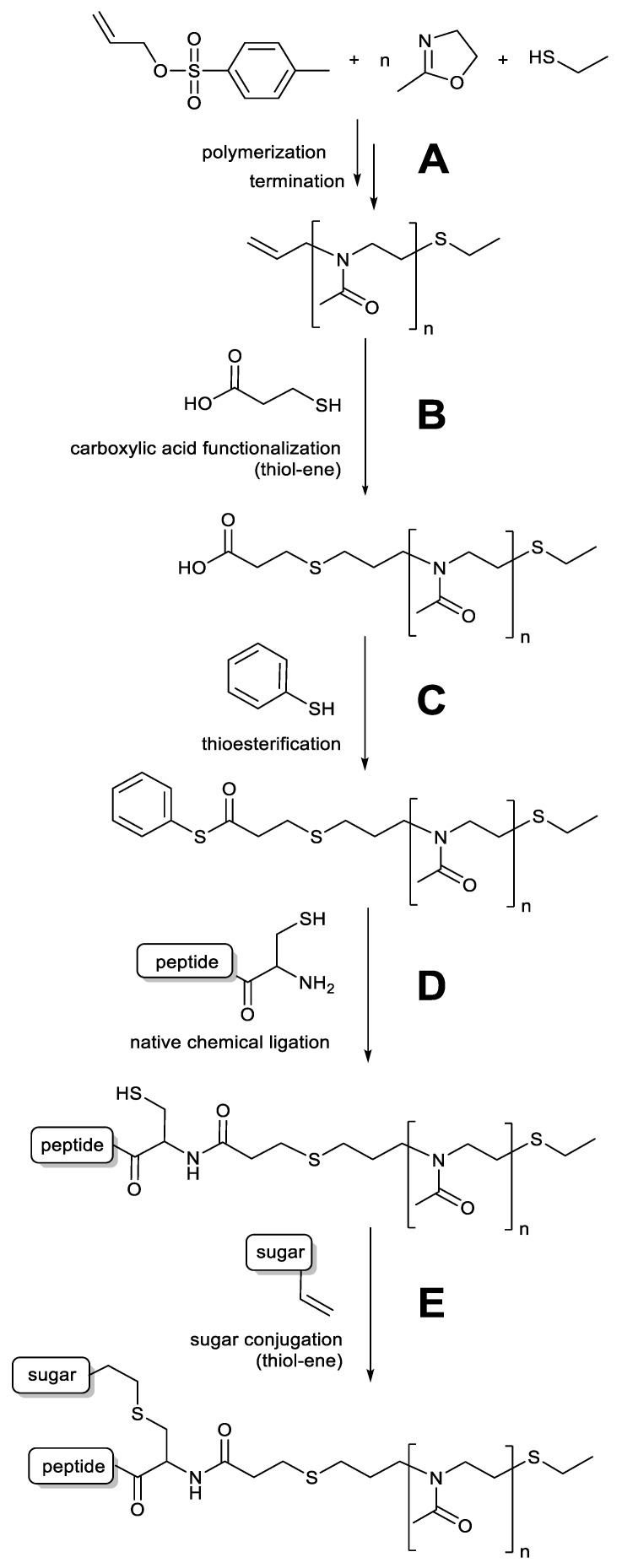
Reaction cascade to functional telechelic polyoxazoline polymers.

The synthesis strategy leading to such a conjugate is depicted in Scheme 1 as a step-by-step reaction cascade. It consists of five steps (A–E), including polymerization and termination leading to the basis allyl-PMeOx_20_-Set polymer (A), attachment of a carboxylic acid functionality (B), conversion into a thioester group (C), NCL for the conjugation of peptides (D), and a thiol-ene-mediated sugar conjugation (E).

#### 3.1.1. Synthesis of Functionalized Polyoxazolines

Telechelic α allyl and ω thioether functionalized PMeOx (step A) was synthesized using allyl *p*-toluenesulfonate as the initiator and ethanethiol as the termination agent in a microwave-assisted polymerization of MeOx.

The initiator allyl *p*-toluenesulfonate was synthesized from allyl alcohol and tosyl chloride in the presence of benzyltrimethylammonium chloride as a stabilizing agent. The reaction was performed for 90 min at 0 °C in a solution of THF with 2.5 M NaOH as a base, as described in the literature. After washing the organic phase with water and removing the solvent, the final product could be obtained in yields of 67%, which is in good accordance with the literature [32,33,34].

For polymerization, the initiator allyl *p*-toluenesulfonate and MeOx as monomers were dissolved in dry acetonitrile. Twenty equivalents of MeOx and one equivalent of initiator were used to achieve an average chain length of 20 repeating units. The polymerization was carried out as a microwave reaction at 100 °C for 60 min under an inert gas atmosphere. In order to introduce the thioether functionality, ethanethiol was used as a nucleophilic terminating agent. It was added to the polymer solution after the microwave reaction and then stirred for 24 h at 40 °C. The polyoxazoline product was purified by precipitation from cold diethyl ether. It will be referred to as allyl-PMeOx_20_-SEt.

**Figure 2:**
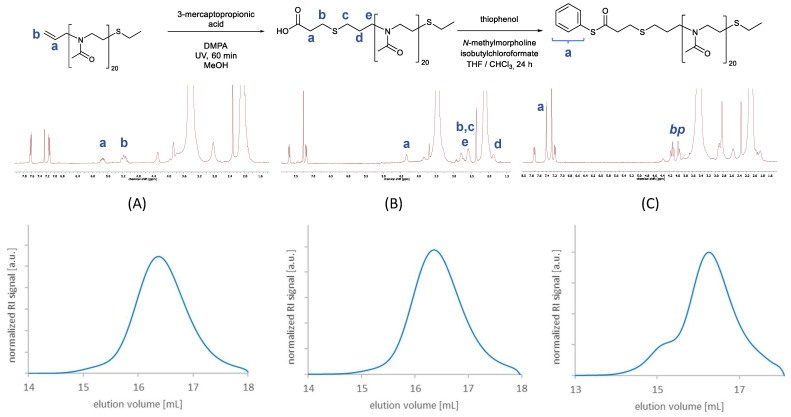
^1^H-NMR (in CDCl_3_) and size exclusion cromatography (in DMF) spectra of allyl (**left**), carboxylic acid (**middle**) and thioesther (**right**) functionalized allyl-PMeOx_20_-SEt (**A**), COOH-PMeOx_20_-SEt (**B**) and COSPh-PMeOx_20_-SEt (bp: unreactive low-molecular by-product, which can easily be removed via dialysis after the next reaction step) (**C**).

The characteristic signals in ^1^H-NMR spectroscopy (see Figure 2, left) at 5.79–5.68 ppm and 5.22–5.07 ppm originate from the allyl group of the initiator and serve as intern reference in order to calculate the degree of polymerization. Clearly recognizable are the strong signals at 3.41 and 2.09–1.99 ppm, which stem from the polymer *backbone* and side chain methyl groups. Integration of these signals delivers a chain length of 20.5 repeating units, which is in good accordance with the amount of 20 equivalents of monomer that were used in the polymerization reaction and in agreement with SEC measurements (see below). The terminating thioether group delivers an alkyl CH_2_ signal at 2.82 ppm.

SEC measurements in DMF showed a narrow molecular weight distribution with a symmetric elugram and a dispersity of *Đ* = 1.05 (see Figure 2, left). The molecular weight determined by SEC (M_n_ = 1759 g·mol^−1^, M_w_ = 1854 g·mol^−1^) corresponds to the molecular weight calculated from ^1^H-NMR spectroscopy (M_n_ = 1847 g·mol^−1^) and is in good accordance with the expected theoretical molecular weight (M_n_ = 1804 g·mol^−1^).

The resulting polymer was then functionalized with a carboxylic acid group via thiol-ene chemistry (step B), using 3-mercaptopropionic acid as a reaction partner and DMPA as photoinitiator. In ^1^H-NMR spectroscopy (see Figure 2 mid), disappearance of the characteristic allyl signals from the educt polymer at 5.84–5.75 and 5.22 ppm and the formation of new signals from the formed alkyl chain as well as alkyl signals from the attached mercaptopropionic acid at 2.94–2.90, 2.78–2.69 and 2.65–2.57 ppm confirm a successful conjugation reaction with a complete conversion of the educt.

SEC measurements in DMF (see Figure 2 mid) show a narrow molecular weight distribution with a symmetric elugram, a dispersity of *Đ* = 1.02 and a molecular weight of M_n_ = 1910 g·mol^−1^, M_w_ = 1960 g·mol^−1^, which is in good accordance with the expected theoretical molecular weight (M_n_ = 1910 g·mol^−1^). Slight tailing effects occur due to interactions of the carboxylic acid group with column material.

IR spectroscopy substantiates a successful reaction by delivering a characteristic signal at 1721 cm^−1^, which is not present in the IR spectrum of the precursor allyl functionalized polymer and stems from the stretching vibration of C=O from the attached carboxylic acid group (for spectrum, see Figure S2 Supplementary Information).

Apart from NMR, IR and SEC measurements, titration of the resulting product against 0.1 M NaOH was also performed to yield information on the conversion rate of the reaction. With a consumption of 1.86 mL NaOH to equivalence point (pH = 6.0) for a 35.2 mg sample, a number of 1.01 COOH groups per polymer chain were determined, which is in good accordance with the theoretically expected value and indicates a complete conversion of the functionalization reaction. The EP was derived from the point where the titration curve had its greatest gradient and was determined from graphic evaluation. For calculation of the functionalization degree, the expected theoretical mass of 1910 g·mol^−1^ was used, which is supported by findings from SEC and NMR spectroscopy (see Figure S3 and Table S1, Supplementary Information, for titration data).

The carboxylic acid group of telechelic functionalized COOH-PMeOx_20_-SEt could be converted into a thioester functionality via thioesterification (step C). For this, COOH-PMeOx_20_-SEt, together with *N*-methylmorpholine as a base, was dissolved in a mixture of chloroform and THF. Isobutyl chloroformate as an activating agent for the carboxylic acid and thiophenol were added, and after purification by precipitation from cold diethyl ether, the resulting thioester functionalized polymer was obtained and will be referred to as COSPh-PMeOx_20_-SEt.

The spectrum also exhibited three signals at 4.25–4.16, 4.00–3.96 and 2.83–2.81 ppm which do not belong to the polymer, but stem from a by-product (bp) that was formed from an isobutyl chloroformate side reaction. This by-product showed similar solubility as the desired polymer and could therefore not be removed by precipitation from cold diethyl ether. Control experiments revealed that formation of the side-product was independent from the thioester formation, because it occurred when only *N*-methylmorpholine, carboxylic acid and isobutyl chloroformate, but no thiol compound, was present. The experiments also proved that it is possible to remove any by-product or unreacted educt via dialysis against water. However, this technique was not utilized to purify the product at this stage of the reaction cascade because it affects the water-sensitive thioester group of the functionalized polymer. The by-product does not affect the NCL reaction; therefore, the raw polymer could be used in subsequent reactions without further purification.

SEC measurements in DMF showed a narrow molecular weight distribution and a molecular weight of M_n_ = 1893 g·mol^−1^, M_w_ = 2142 g·mol^−1^, which is in good accordance with the expected theoretical molecular weight (M_n_ = 2003 g·mol^−1^). The spectrum exhibited a weak high molecular shoulder as well as a low molecular plateau in the elugram, which, as also described elsewhere [35,36,37,38,39], is attributed to aggregation effects. These effects were also deemed to be responsible for a slightly increased dispersity of *Đ* = 1.13 compared to the precursor COOH-PMeOx_20_-SEt polymer.

IR spectroscopy confirmed a successful conversion of the carboxylic acid group into a thioester group, which was observable through a wavelength shift from 1721 cm^−1^ to 1703 cm^−1^ of the respective characteristic C=O stretching vibration signal (for spectrum, see Figure S4, Supplementary Information).

#### 3.1.2. Synthesis of Two-Part Polymer Peptide Conjugates via NCL

The telechelic functionalized thioester PMeOx_50_-COSPh polymer was conjugated with two different peptides, CGGGF and CGGWYKYW in an NCL (step D). A cysteine functionality is necessary for the NCL; therefore, each of the peptides bears an *N*-terminal cysteine. Both peptides were ordered and synthesized from *GeneCust*. Compared to the work of Markey et al. [40], who describe a similar set of reactions for the conjugation of peptides, this work focuses on the conjugation of polymers as starting materials.

For the reaction, the peptide and COSPh-PMeOx_20_-SEt were dissolved in methanol under the presence of TCEP as a reducing agent and the reaction was stirred for four days. In the case of CGGWYKYW, elevated reaction temperatures of 65 °C were necessary to prevent gel formation. The resulting peptide functionalized polymers will be referred to as CGGGF-PMeOx_20_-SEt, resp. CGGWYKYW-PMeOx_20_-SEt and were obtained and purified via precipitation from cold diethyl ether and dialysis against water (MWCO: 1000 g·mol^−1^) for four days.

**Figure 3:**
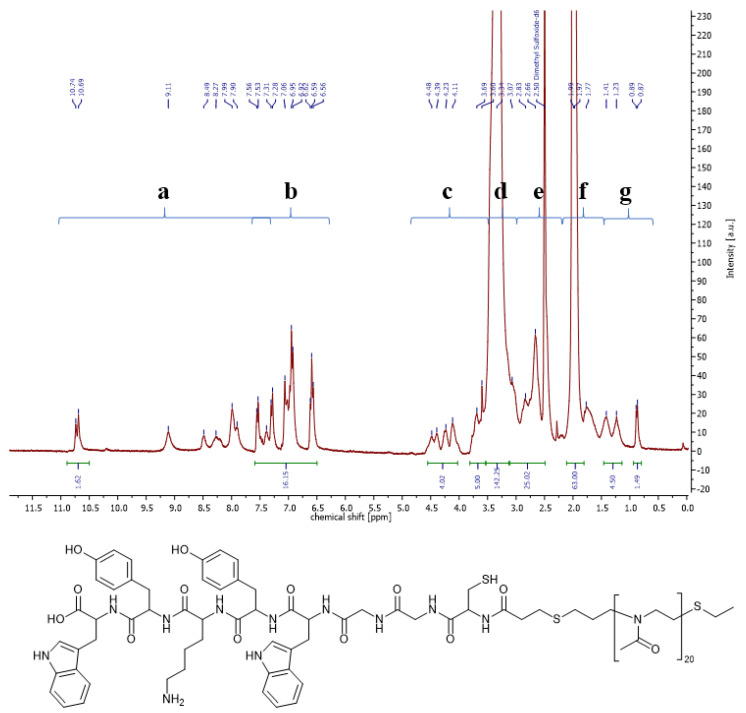
^1^H-NMR spectrum of telechelic peptide functionalized CGGWYKYW-PMeOx_20_-SEt in deuterated DMSO. The spectrum is subdivided into different regions: “NH and NH_2_ region” (**a**); “aromatic region” (**b**); “peptide backbone region” (**c**); “polymer backbone” (**d**); “side chain alkyl region” (**e**); “polymer side chain region” (**f**); and “lysine region” (**g**).

Figure 3 shows a representative ^1^H-NMR spectrum of CGGWYKYW-PMeOx_20_-SEt. The spectrum displays both characteristic signals from the peptide as well as the polymer. Clearly recognizable are some individual signals such as the tryptophan indole NH at 10.72 ppm, the aromatic ring signals of tyrosine and tryptophan at 7.56–6.56 ppm from the peptide part of the conjugate, or the methyl side chain group at 1.99–1.97 ppm from the polymer part of the conjugate. With so many protons contributing to the final spectrum and superimposing each other, it is not feasible to assign the rest of the signals individually. Instead of assigning and integrating single proton signals, the spectrum was subdivided into different regions (a–g). The total integral values that were determined for each region matched the expected number of protons of each part of the conjugate.

IR spectroscopy measurements were also performed with the conjugate (see Figure S5, Supplementary Information). Compared to the educt polymer spectrum, a characteristic signal from the N–H amide bond bending vibration of the peptide was visible at 1531 cm^−1^ (CGGGF) resp. 1515 cm^−1^ (CGGWYKYW) in the product spectrum after conjugation. While the mere educt polymer spectra show no signal at this position, the polymer–peptide conjugate spectra display an apparent peak. The same accounts for the N–H stretching vibration at 3282 cm^−1^. The spectra were taken after dialysis of the product; therefore, the findings affirm conjugation of the peptide to the polymer.

SEC measurements were also performed for all peptide polymer conjugates in water as well as in DMF as solvent. However, it is known from the literature that, especially in the case of peptide conjugates, interactions between substrate and column material can be so strong that either none or else only strongly deviating signals of the substrate can be detected [41,42]. In our case, those effects were so strong that the peptide was retained in the chromatography column. Therefore, SEC measurements did not yield any reliable results, and characterization of the respective conjugates was constrained to classical spectroscopic measurements as well as detailed studies of its interaction behavior on the sensor surface.

#### 3.1.3. Synthesis of Three-Part Polymer Sugar Conjugates via Thiol-Ene Reaction

The telechelic peptide polymer conjugates were conjugated with two different saccharide molecules, protected acetyl allyl α mannose (AcMan) and deprotected allyl α mannose (Man) via a thiol-ene reaction (step E). Peracetylation of mannose and successive BF_3_*Et_2_O mediated functionalization with allyl alcohol provided AcMan in good yields of 70%. Cleavage of the acetyl protective groups via sodium methanolate yielded α-O-allyl-mannoside quantitatively. The thiol functionality of the conjugate, which is necessary for the thiol-ene reaction, is formed through the preceding NCL reaction.

Both educts, the respective polymer peptide conjugate and sugar, were dissolved in water in the presence of TCEP to guarantee reductive conditions which prevented the free thiol group from dimerizing. In the case of CGGWYKYW-PMeOx_20_-SEt, small amounts of DMSO were added to the reaction mixture to facilitate solubility. Irgacure 2959 was chosen as an initiator because of its better water solubility (compared to DMPA), and the reaction mixture was stirred for 60 min under UV light irradiation. The resulting peptide and carbohydrate-functionalized polymer conjugates will be referred to as Man-CGGGF-PMeOx_20_-SEt, Man-CGGWYKYW-PMeOx_20_-SEt and AcMan-CGGWYKYW-PMeOx_20_-SEt and were purified via dialysis against water (MWCO: 1000 g·mol^−1^) for four days. Over a timespan of a minimum of two months, the final conjugates showed no instabilities or degradation effects in the respective solvents.

The ^1^H-NMR spectroscopy indicated that conjugation of α-O-allyl-mannoside with the polymer was also successful, because no more allyl signals were present in the product spectrum at 5.93–5.84 ppm and there was a newly formed characteristic signal at 5.10 ppm that corresponded to the attached mannose unit. It could be found in a region of the spectrum where no other signals were present in the precursor conjugate spectrum (see Figure S6, Supplementary Information).

**Figure 4:**
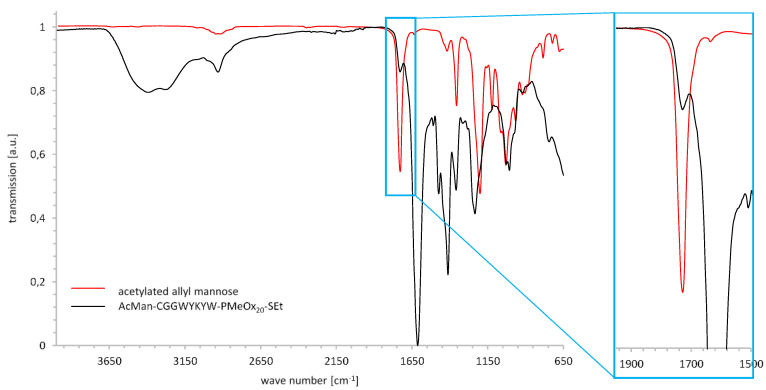
Superimposed IR spectra of acetylated allyl mannose (red) and product conjugate AcMan-CGGWYKYW-PMeOx_20_-SEt (black) with weak, but characteristic, C=O stretching vibration at 1736 cm^−1^ (highlighted with blue box).

IR spectroscopy was performed in order to adduce evidence for the accomplished conjugation. The product IR spectrum (see Figure 4) exhibits a characteristic signal for C=O stretching vibration of the sugar acetyl groups at 1736 cm^−1^, which is not present in the educt IR spectrum, and therefore confirms findings from the NMR spectroscopy.

In contrast to conjugates with acetylated allyl mannose, deprotected allyl mannose exhibited no distinctive, characteristic signals in IR or NMR spectroscopy. It only delivered signals in an area of the ^1^H-NMR spectrum where they were easily overlaid by many CGGWYKYW peptide and polymer backbone signals. Consequently, the most important sign for a successful reaction is the disappearance of the allyl signals at 5.98–5.15 ppm, which was already proven to be a good indication for conversion in previous reactions. Additionally, minor sugar signals at 3.97–3.84 ppm and an increase in integral values could be detected, as well as a singlet signal at 4.65 ppm which was not present in the precursor spectrum and was assigned to the anomeric proton of the sugar ring (for spectrum see Figure S8, Supplementary Information).

### 3.2. Biomacromolecular Recognition Experiments

The success of all cascade reaction steps leading to the final conjugation product was proven through biomacromolecular recognition experiments with the lectin ConA. Two different methods for the analysis of substrate binding affinities were chosen to investigate the conjugates at hand: SAW and SPR measurements.

#### SAW Affinity Studies

In SAW measurements, binding affinities of substrates are measured via alteration of the phase, amplitude, and frequency of surface acoustic waves in case of a binding event.

SAW measurements consist of three steps. At first, the polymer or polymer conjugate is attached to the gold surface of the sensor chip by incubating three of the available five measuring lanes with polymer solution. The two remaining empty lanes serve as blind test lanes. Next, the non-covered surface area is saturated with bovine serum albumin (BSA). This step, the saturation with BSA, already yields the first information concerning the binding of a polymer or polymer conjugate to the surface. For the blind lanes, a high phase change is measured, whereas for gold lanes that are covered with polymer, the binding of BSA is significantly lower (see Figure S10, Supplementary Information for spectra). No difference in the BSA saturation experiments is observed between different polymer conjugates.

Following the preparation of the gold surface with the polymer conjugates and saturation with BSA, the main measurement can be performed by analyzing the binding affinity of the lectin to the functionalized surface. For this, a constant flow of a series of ConA dilutions with defined concentrations of ConA is injected on each channel and the phase change of surface acoustic waves, which are influenced by binding events, is monitored. If there is no binding event, no change in phase can be detected. In the case of a binding event, the phase curve rises as long as there is a constant flow of lectin solution over the measuring lanes until it reaches a saturation plateau after a few minutes. ConA is not covalently bound to the conjugates; therefore, as soon as the flow of lectin stops, the curve declines as a result of a detachment event of ConA from the surface. Between the measurements with different concentrations of ConA, all lanes are regenerated with an acidic glycine solution to detach all remaining bound proteins. After measuring four different ConA concentrations, the binding and dissociation curves are fitted and the K_D_ value is calculated.

**Figure 5:**
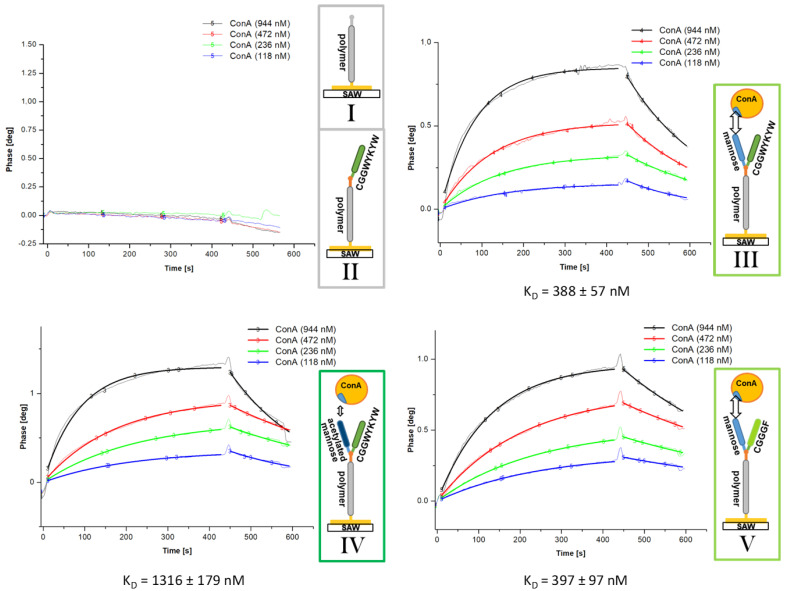
Exemplary SAW measurement curves for experiments I–V. For each experiment, four different lectin concentrations were measured. Experiments I and II (**top-left**) did not show any binding events.

As shown in Figure 5, five different experimental settings for the interaction of polymer conjugates with ConA were investigated.

In experiment I, an unfunctionalized polymer was attached to the surface and served as a negative control. Affinity measurements showed no binding event of ConA to the surface, which is as expected (see Figure 5, top left).

There is no indication, either in literature or from preceding experiments, for a specific interaction of ConA to peptides with a CGGWYKYW sequence. Thus, the conjugate of polymer and CGGWYKYW is also not expected to show affinity to ConA, which could be proven in experiment setting II. The measurement curves look similar to experiment I and no binding event was observed.

In contrast, mannose derivatives are expected to have a high affinity to ConA [43,44]. Conjugates between polymer and mannose therefore showed strong binding to ConA. This was examined in experiment III (see Figure 5, top right), where a conjugate between polymer, peptide CGGWYKYW, and mannose sugar was attached to the gold surface. An affinity to the lectin with a K_D_ of 388 ± 57 nM could be measured. The experiment was repeated several times with different batches of polymer conjugate, and each time yielded identical results with similar K_D_ values.

Excluding a cross reactivity to lectins other than ConA was shown by repeating experiment I–III with Gal1 as a binding target. The respective experiments displayed no affinity between Gal1 and the polymer conjugates.

Modifying the sugar unit has an influence on the binding affinity to the lectin. In experiment IV (see Figure 5, bottom-left), a similar conjugate as in experiment III was examined, but the mannose unit attached to the polymer was protected with acetyl groups. In this case, there was still an affinity to the lectin. However, with a K_D_ value of 1316 ± 179 nM, the binding of ConA to the surface was significantly weaker than before, because the acetyl groups prevented an optimal fit of the mannose unit into the binding pocket of ConA.

In setting V, the CGGWYKYW peptide was exchanged with a different peptide to show that the choice of peptide did not influence sugar binding affinity. CGGGF did not show specific interaction with ConA, and the K_D_ value of 397 ± 97 nM for this experiment is comparable to experiments with CGGWYKYW (see Figure 5, bottom-right).

### 3.3. SPR Affinity Studies

SPR spectroscopy is a standard method for measuring the adsorption of material onto surfaces or nanoparticles and relies on the excitation of surface plasmons by polarized light, irradiated through a prism. Changes of the refractive index of a surface due to binding events are measured.

**Figure 6:**
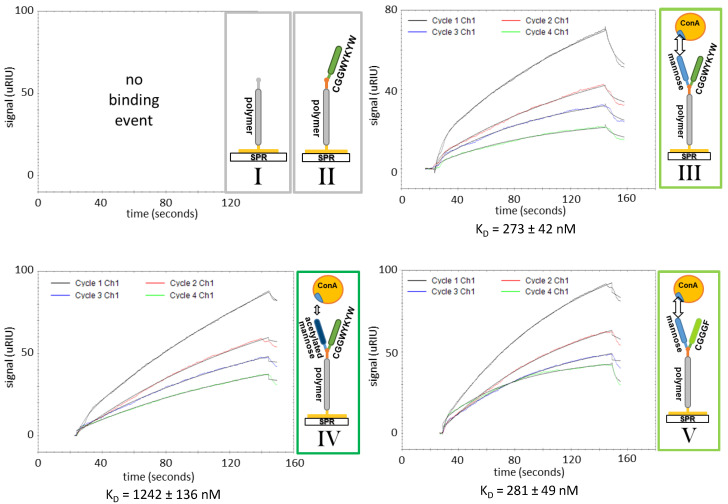
Exemplary SPR measurement curves for experiments I–V. For each experiment, four different lectin concentrations were measured. Experiments I and II (**top-left**) did not show any binding events.

SPR spectroscopy was performed as an ancillary analysis that supported the findings from SAW measurements. Preparing the samples is identical to SAW measurements. The SPR measuring channels were coated with polymer solution, and unoccupied gold surface was saturated with BSA. The measuring curves of the binding event of ConA to the surface had a similar shape as those measured by SAW (see Figure 6).

All results from SPR and SAW spectroscopy are listed in Table 1. The SPR measurements deliver K_D_ values that comply with the results obtained by SAW measurements. For unfunctionalized allyl-PMeOx_20_-SEt and peptide polymer conjugate CGGWYKYW-PMeOx_20_-SEt, no binding event was monitored. For conjugates with mannose, there is no significant difference in K_D_ values between conjugates with different peptides. The conjugates exhibit K_D_ values of 250 ± 34 nM for Man-CGGWYKYW-PMeOx_20_-SEt and 255 ± 40 nM for Man-CGGGF-PMeOx_20_-SEt. With a K_D_ value of 1138 ± 193 nM, the conjugate of acetyl protected mannose displays a significantly weaker binding to ConA than its deprotected derivate. Overall, the K_D_ values determined by SPR spectroscopy are in the same order of magnitude as the K_D_ values determined by SAW measurements.

## 4. Discussion

In conclusion, a versatile toolbox for the design of bioactive three-part conjugates, suitable for biomacromolecular recognition events in a self-assembled monolayer measuring setup, was developed by the chemoselective and vicinal coupling of two biological components to the binding site at one telechelic end of a double functionalized POx polymer chain. An α allyl as well as ω thioether functionalized polyoxazoline was first synthesized as a polymer starting material. The succeeding steps included a modification of the allyl end group to carboxylic acid before converting it into a thioester group via thioesterification. Next, two different cysteine functionalized peptides CGGGF and CGGWYKYW were successfully conjugated to the thioester functionalized polymers in an NCL, and the attachment of two different sugar derivates, acetylated mannose as a model saccharide as well as a deprotected mannose unit, was successful via the use of a thiol-ene click reaction. The success of the reaction cascade was further verified by ensuing biomacromolecular recognition SAW experiments, and results from supplementary SPR spectroscopy reinforce those findings. POx-conjugated deprotected mannose showed a binding affinity to ConA with a K_D_ of 351 ± 77 nM (SAW), while protected mannose showed significantly weaker binding affinities with a K_D_ of 1325 ± 97 nM (SAW). As expected, the peptide residue had no influence on the K_D_ value, which was proven in experiments with different pendant peptide moieties.

Together with the final affinity measurements that frame the whole cascade by positively proving the last of multiple reaction steps, this work contributes to overcoming the challenges of the design of precise and defined polymer conjugates. At this point, it also seems conceivable to transfer this established set of reactions to other specific and biologically relevant peptides and sugars that exhibit distinct interactions with tissues or proteins, providing a simple way to reliably determine biological interactions and corresponding affinity values of multivalent materials.

## Figures and Tables

**Table 1:** K_D_ values of SAW and SPR affinity measurements. Each experiment was conducted on three (SAW) resp. four (SPR) lanes with four different lectin concentrations being measured on each lane. The average K_D_ value resulting from all individual measurements combined is highlighted in bold (no aff. means no affinity of the conjugate to the lectin could be determined).

		SAW K_D_ [nM]			SPR K_D_ [nM]			
		Lane 1	Lane 2	Lane 3	Lane 1	Lane 2	Lane 3	Lane 4
**allyl-PMeOx_20_-SEt (I)**		**no aff.**			**no aff.**			
**CGGWYKYW-PMeOx_20_-SEt (II)**		**no aff.**			**no aff.**			
**Man-CGGWYKYW-PMeOx_20_-SEt (III)**		**351 ± 77**			**250 ± 34**			
	(1)	257 ± 60	388 ± 92	383 ± 46	214 ± 32	229 ± 38	273 ± 42	285 ± 46
	(2)	N/A	329 ± 42	430 ± 34	-			
	(3)	278 ± 44	432 ± 71	467 ± 62	-			
	(4)	303 ± 75	388 ± 57	215 ± 25	-			
**AcMan-CGGWYKYW-PMeOx_20_-SEt (IV)**		**1325 ± 97**			**1138 ± 193**			
		1316 ± 179	1426 ± 293	1232 ± 266	894 ± 117	1081 ± 83	1242 ± 136	1336 ± 126
**Man-CGGGF-PMeOx_20_-SEt (V)**		**332 ± 63**			**255 ± 40**			
		272 ± 21	328 ± 55	397 ± 97	234 ± 41	204 ± 22	281 ± 49	293 ± 53

## Data Availability

Further data is available from the authors on request.

## Supplementary Materials

The following are available online at https://www.mdpi.com/article/10.3390/s21093153/s1, Figure S1: ^1^H-NMR spectrum of allyl *p*-toluenesulfonate in CDCl_3_; Figure S2: Superimposed IR spectra of educt allyl functionalized polymer and product carboxylic acid functionalized polymer; Figure S3: Titration curve of COOH-PMeOx_20_-SEt; Figure S4: Titration curve of COOH-PMeOx_20_-SEt; Figure S5: Superimposed IR spectra of educt polymer COSPh-PMeOx_20_-SEt (grey), product polymer peptide conjugate CGGWYKYW-PMeOx_20_-SEt (black) and peptide CGGWYKYW (green); Figure S6: Stacked ^1^H-NMR spectra of educt CGGWYKYW-PMeOx_20_-SEt (grey, top) in deuterated DMSO, educt acetylated allyl mannose (blue, middle) in CDCl_3_ and the product AcMan-CGGWYKYW-PMeOx_20_-SEt conjugate (red, bottom) in deuterated DMSO; Figure S7: ^1^H-NMR spectrum of Man-CGGWYKYW-PMeOx_20_-SEt after dialysis in deuterated DMSO; Figure S8: Section of the Man-CGGWYKYW-PMeOx_20_-SEt product spectrum (blue) where sugar signals are visible; Figure S9: ^1^H-NMR spectrum of Man-CGGGF-PMeOx_20_-SEt after dialysis in MeOD; Figure S10: Exemplary spectra of the saturation of the gold surface with BSA in SAW affinity studies; Table S1: pH titration of carboxylic acid functionalized polymers against 0.01 M sodium hydroxide solution.
