# Supplementary material for: Polyoxazolines with a Vicinally Double-Bioactivated Terminus for Biomacromolecular Affinity Assessment
Source: Sensors (Basel). 2021 May 1;21(9):3153. doi: 10.3390/s21093153 (PMC8125408; doi:10.3390/s21093153)
Supplement: Supplementary file 1 [file sensors-21-03153-s001.zip › sensors-1127191-supplementary.pdf]

*Supporting Information*

# Polyoxazolines with Vicinally Double-Bioactivated Terminus for Biomacromolecular Affinity Assessment

**Florian Pinzner [1], Jürgen Mut [2], Thorsten Keller [1], Julian Bechold [2], Jürgen Seibel [2] and Jürgen Groll [1,*]**

1. Department of Functional Materials in Medicine and Dentistry, Institute of Functional Materials and Biofabrication and Bavarian Polymer Institute, University of Würzburg, Pleicherwall 2, 97070 Würzburg, Germany.
2. Institute of Organic Chemistry, University of Würzburg, Am Hubland, Würzburg, Germany.

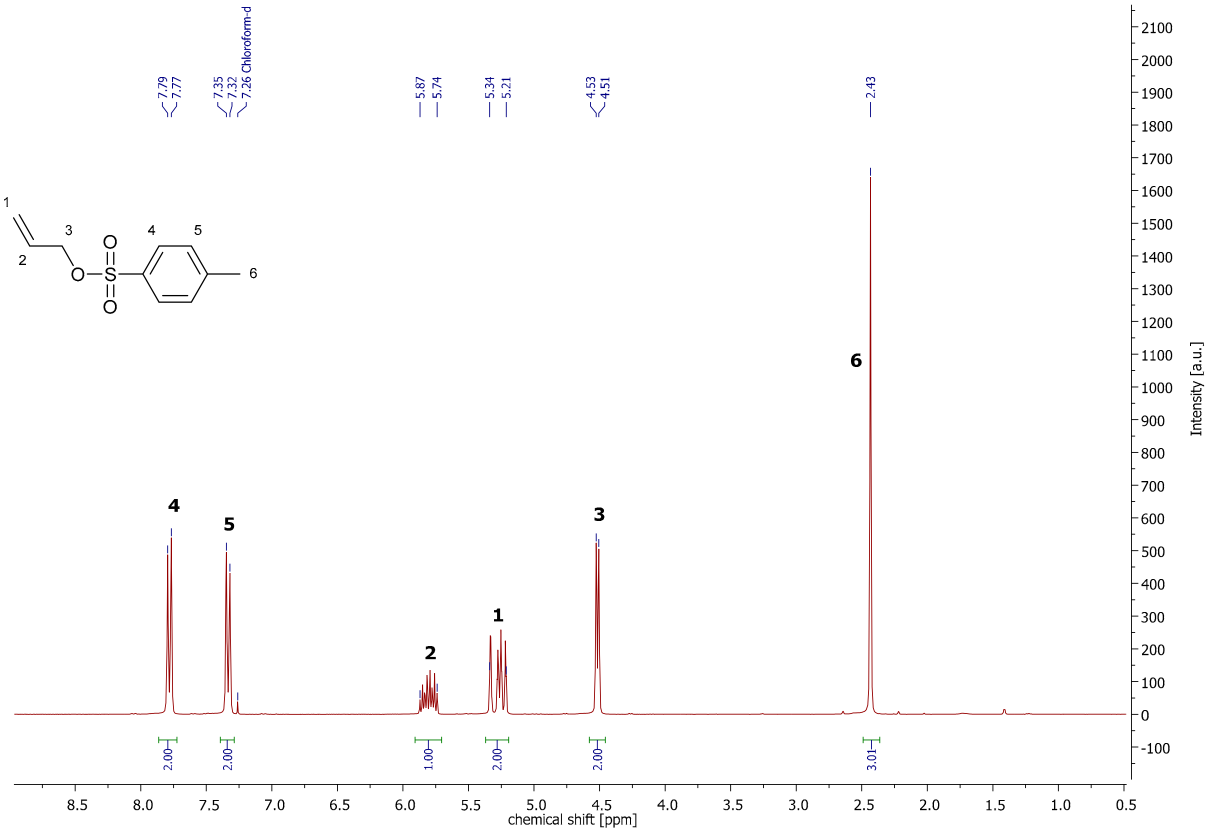


**Figure S1.** $^1$H-NMR spectrum of allyl p-toluenesulfonate in $CDCl_3$.

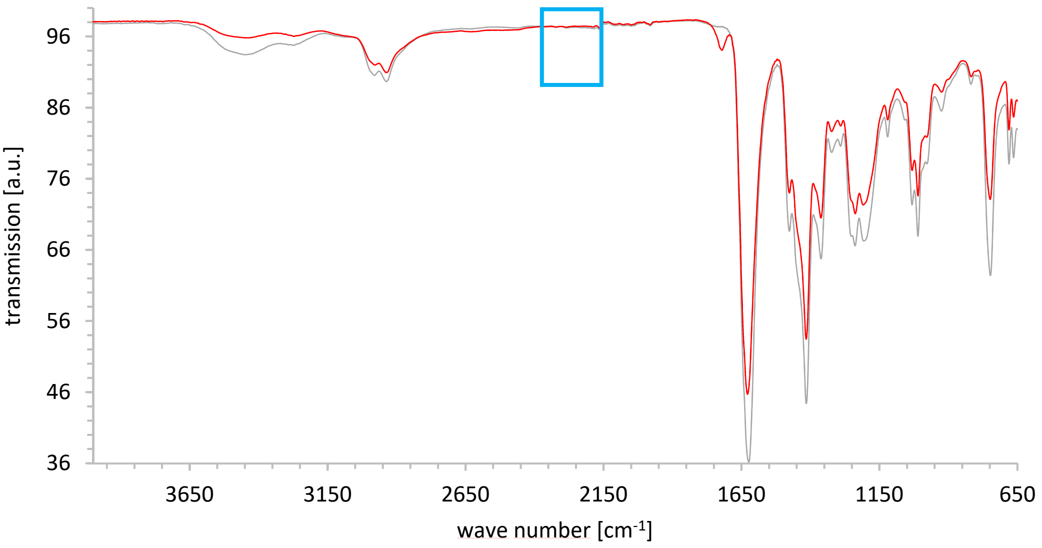


**Figure S2.** Superimposed IR spectra of educt allyl functionalized polymer allyl-PMeOx$_{20}$-SEt (grey) and product carboxylic acid functionalized polymer COOH-PMeOx$_{20}$-SEt (red) with characteristic C=O stretching vibration of the carboxylic acid group at 1721 cm$^{-1}$ (highlighted with blue box).

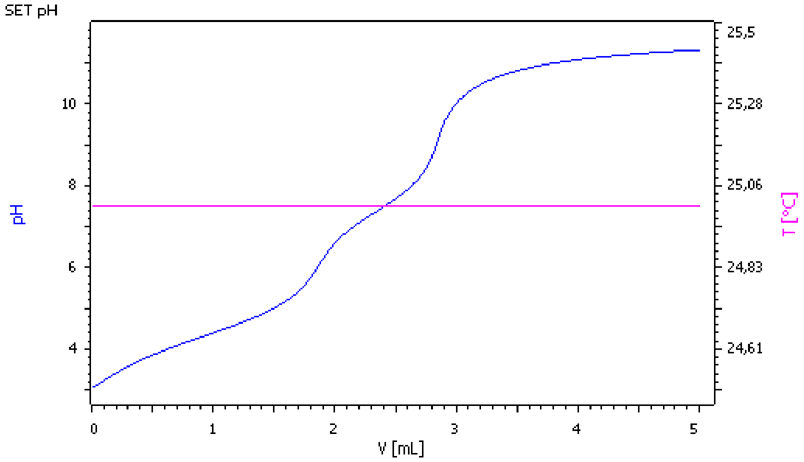


**Figure S3.** Titration curve of COOH-PMeOx$_{20}$-SEt.

**Table S1.** pH titration of carboxylic acid functionalized polymers against 0.01 M sodium hydroxide solution.

| $M_n$ theo. [g·mol$^{-1}$] | sample weight m [mg] | NaOH consumption V (NaOH) (0.01M) to pH7 [mL] | pH equivalence point* | NaOH consumption to equivalence point (EP) [mL] | number of COOH groups per polymer chain (theo.) | number of COOH groups per polymer chain ** | number of COOH groups per polymer chain (exp.)*** |
|---|---|---|---|---|---|---|---|
| 1910 | 35.2 | 2.157 | 6.0 | 1.858 | 1 | 1.17 | **1.01** |

*determined by graphic evaluation, **calculated by NaOH consumption to neutral point (NP, pH=7),.***calculated by NaOH consumption to first equivalence point (EP).

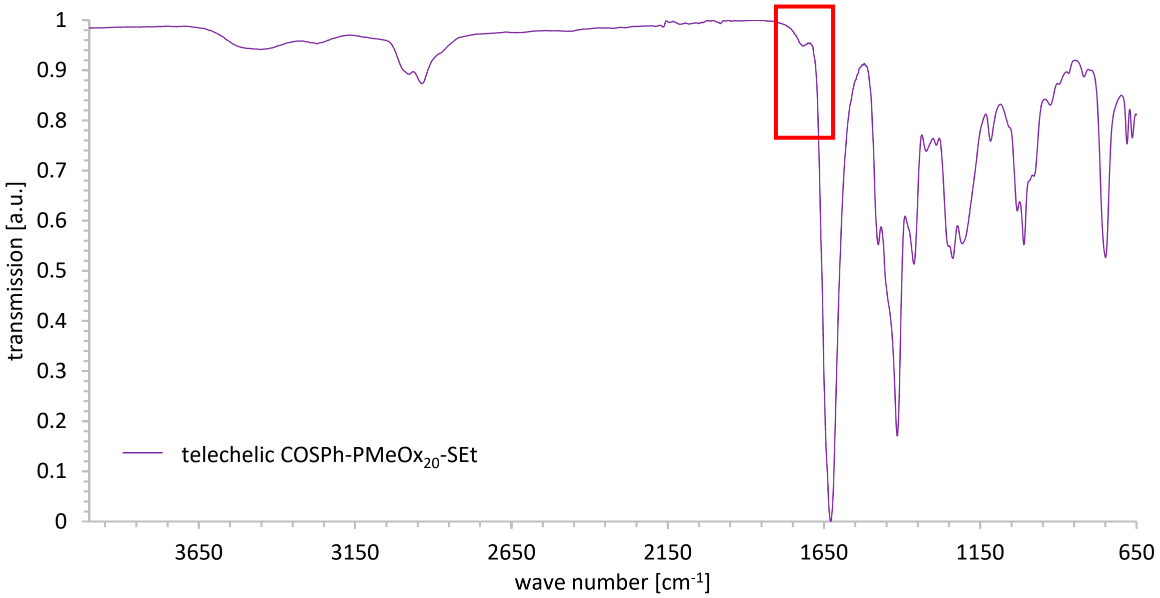


**Figure S4.** IR spectra of telechelic thioester functionalized polymer COSPh-PMeOx20-SEt with weak, but characteristic C=O stretching vibration of the thioester group at 1703 cm$^{-1}$ (highlighted with red box).

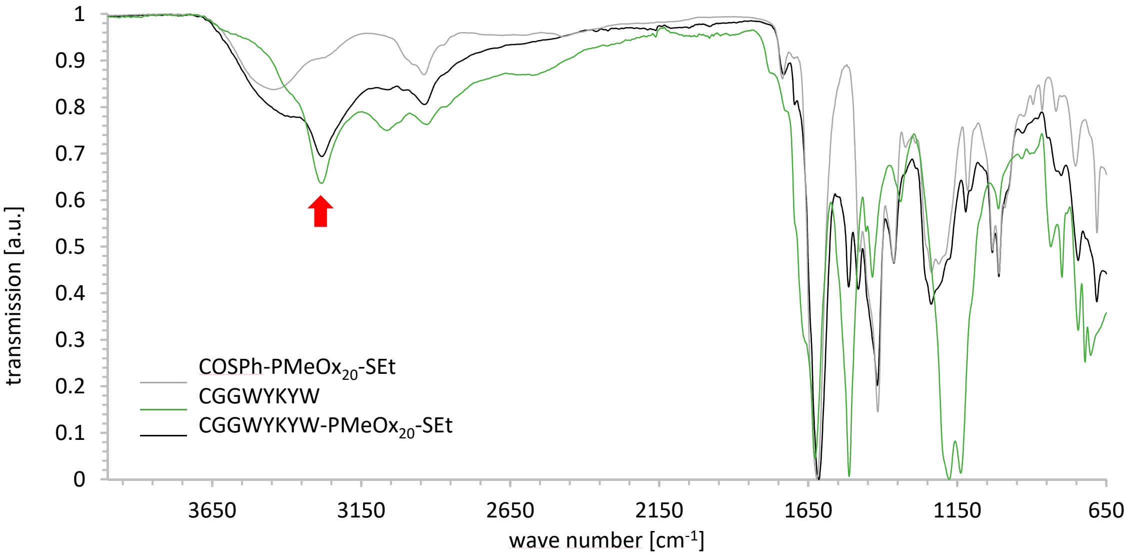


**Figure S5.** Superimposed IR spectra of educt polymer COSPh-PMeOx20-SEt (grey), product polymer peptide conjugate CGGWYKYW-PMeOx20-SEt (black) and peptide CGGWYKYW (green) with characteristic N-H stretching vibration and amide bond bending vibration of CGGWYKYW at 3282 and 1515 cm$^{-1}$ (highlighted with red arrows). The relevant part of the spectrum is enlarged.

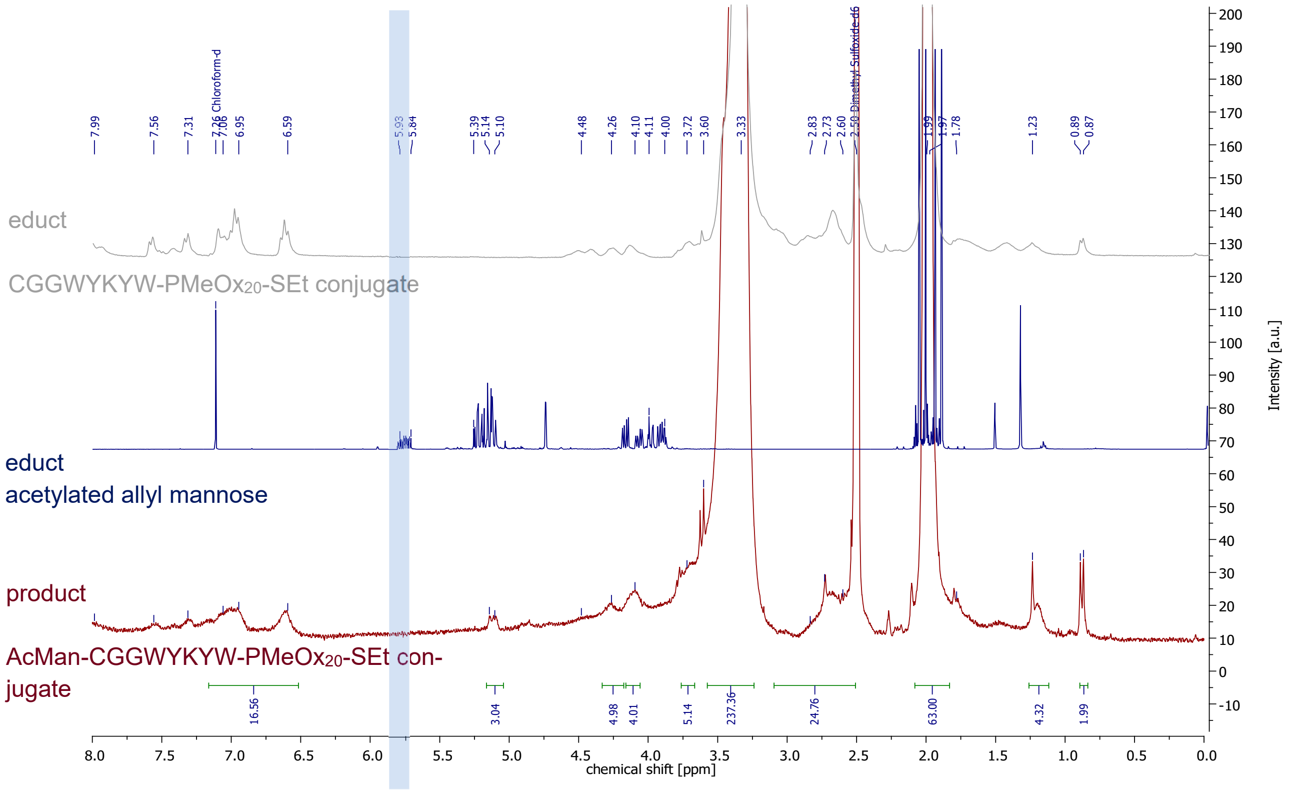


**Figure S6.** Stacked $^{1}$H-NMR spectra of educt CGGWYKYW-PMeOx20-SEt (grey, top) in deuterated DMSO, educt acetylated allyl mannose (blue, middle) in $CDCl_3$ and the product AcMan-CGG-WYKYW-PMeOx20-SEt conjugate (red, bottom) in deuterated DMSO with the most relevant signal highlighted in blue.

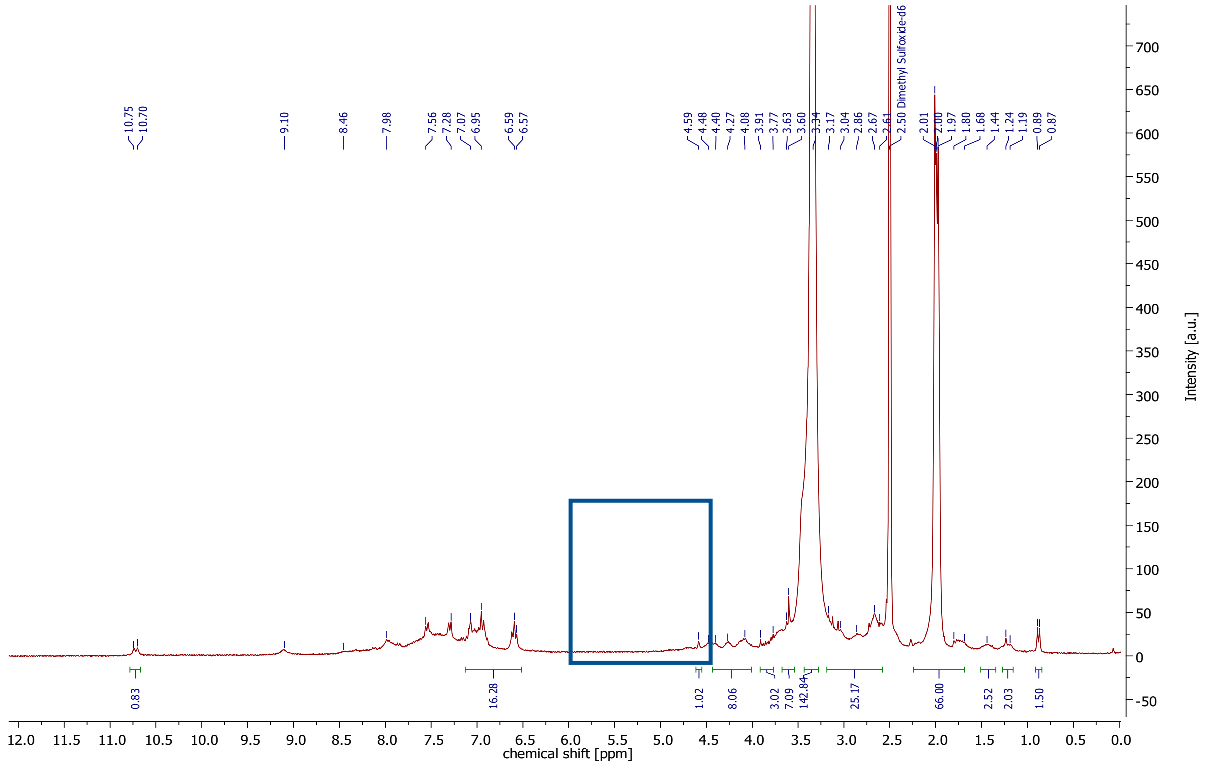


**Figure S7.** $^{1}$H-NMR spectrum of Man-CGGWYKYW-PMeOx20-SEt after dialysis in deuterated DMSO.

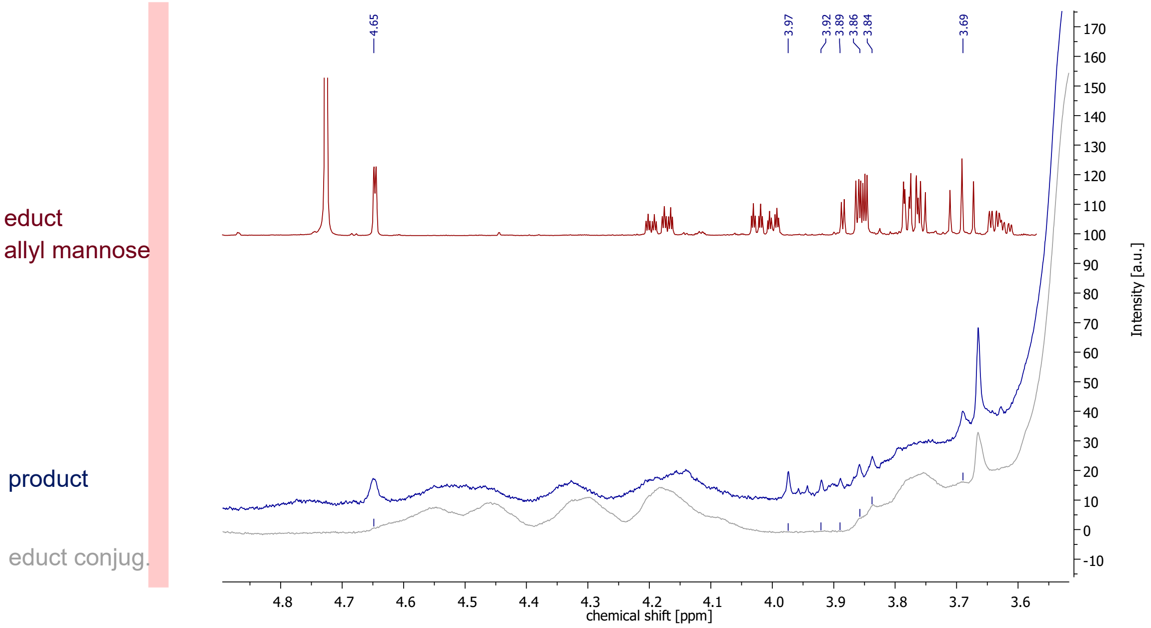


**Figure S8.** Section of the Man-CGGWYKYW-PMeOx$_{20}$-SEt product spectrum (blue) where sugar signals are visible. The anomeric proton is highlighted in red. The spectrum is stacked with the precursor spectrum (grey) and the educt allyl mannose spectrum (red).

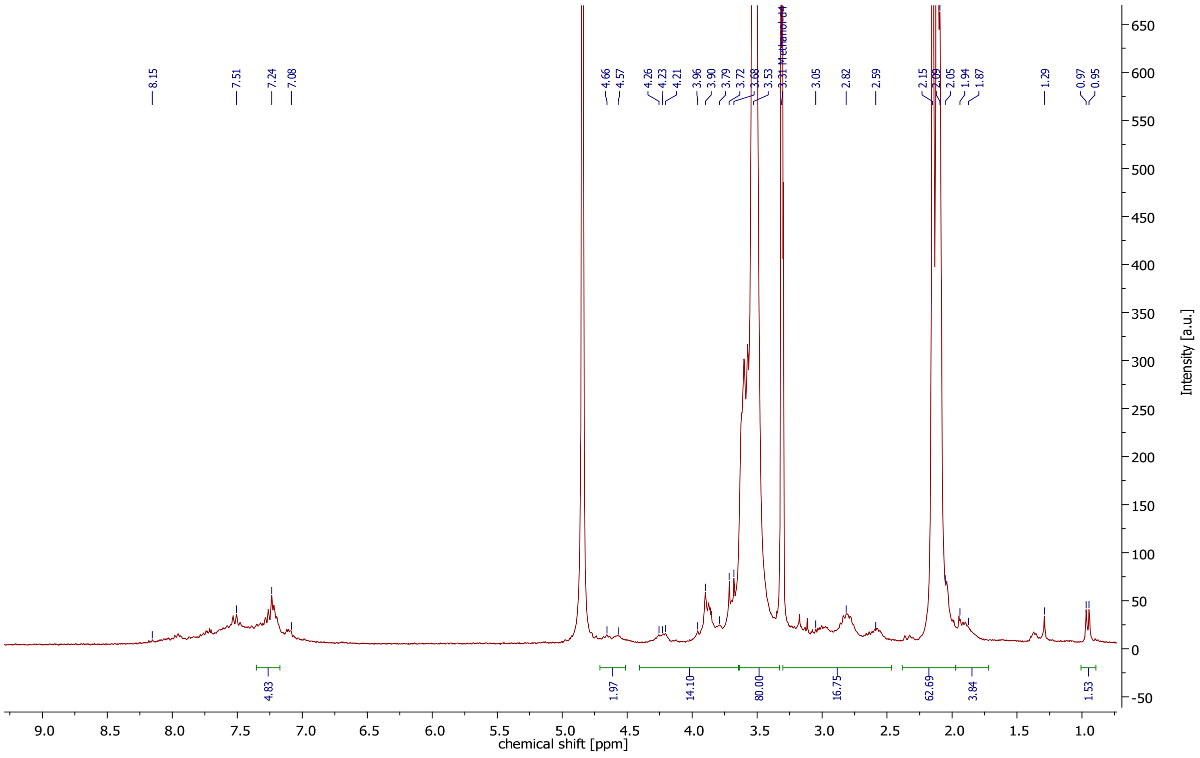


**Figure S9.** $^1$H-NMR spectrum of Man-CGGGF-PMeOx$_{20}$-SEt after dialysis in MeOD.

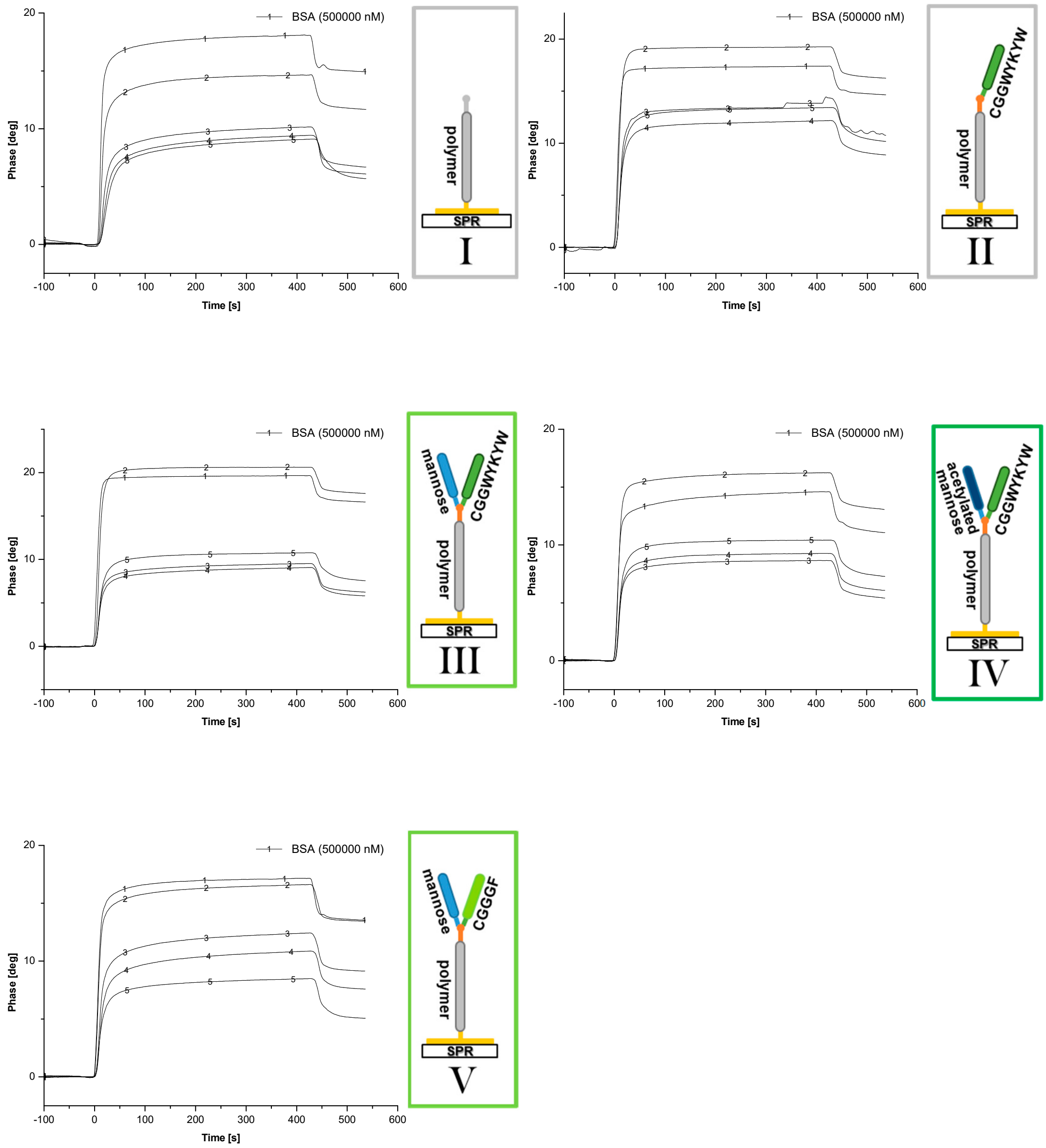


**Figure S10.** Exemplary spectra of the saturation of the gold surface with BSA in SAW affinity studies. In each experiment, the blind test lanes 1 and 2 (top curves) have not been previously coated with polymer and therefore allow for more binding of BSA. This effect is observed similarly with all different polymer conjugates I-V..
